# Comparative Assessment of Lignan, Tocopherol, Tocotrienol and Carotenoids in 40 Selected Varieties of Flaxseed (*Linum usitatissimum* L.)

**DOI:** 10.3390/foods12234250

**Published:** 2023-11-24

**Authors:** Zhimin Wu, Yazhi Li, Huajiao Qiu, Songhua Long, Xinlin Zhao, Yufu Wang, Xinbo Guo, Aliya Baitelenova, Caisheng Qiu

**Affiliations:** 1 Institute of Bast Fiber Crops and Center of Southern Economic Crops, Chinese Academy of Agricultural Sciences, Changsha 410205, China; wuzhimin@caas.cn (Z.W.); 82101225153@caas.cn (Y.L.); qiuhuajiao@caas.cn (H.Q.); longsonghua@caas.cn (S.L.); zhaoxinlin@caas.cn (X.Z.); wangyufu@caas.cn (Y.W.); 2 School of Food Science and Engineering, South China University of Technology, Guangzhou 510641, China; guoxinbo@scut.edu.cn; 3 Department of Plant Production, Faculty of Agronomy, S.Seifullina Kazakh Agrotechnical Research University, Astana 010000, Kazakhstan; baitelenova_alya@mail.ru

**Keywords:** flaxseed, lignan, tocopherol, tocotrienol, carotenoid, variety comparison

## Abstract

Multiple varieties of flaxseeds have been identified in the world, yet the relationship between these varieties, their agronomic traits, and their seeds’ quality remains unclear. This study aimed to determine the level of lignan, vitamins and carotenoids in 40 selected flaxseed varieties, and the relationship between varieties, agronomic traits, and seed quality was investigated. In this study, notably, fiber flax variety No. 225 exhibited the highest lignan content among all tested seeds. Additionally, oil variety No. 167 demonstrated the highest level of α-tocotrienol (α-T3), β-tocopherol (β-T), γ-tocotrienol (γ-T3), and β-carotene (β-Car.). Conversely, intermediate flax variety No. 16 displayed the highest content of α-tocopherol (α-T), but lowest content of lutein (Lut.), zeaxanthin (Zea.), β-carotene (β-Car.), and total carotenoids (Total Car.). Furthermore, a correlation was observed between petal color with the lignan, while a strong correlation has been explored in seed yield, seed type, plant natural height, and fiber content in straw. Nevertheless, further investigation is required to elucidate the internal relationship between varieties with compositions.

## 1. Introduction

Flaxseed, a fundamental agricultural and industrial raw material produced for human consumption, contains high levels of bioactive compounds such as lignan and vitamins [1]. It serves not only as a major food resource but also as an important bioreactor for the production and storage of phytochemicals [2,3,4]. As a nutritional resource, flax possesses a beneficial chemical composition in oil, characterized by abundant unsaturated fatty acids, soluble fiber, protein, lignan, vitamins, carotenoid, and antioxidants (Figure S1) [5]. Furthermore, flaxseed is considered as an attractive source of lignan and other nutrients such as carotenoids and vitamin E, and was associated with the control of diabetes [6], alleviation of cardiovascular diseases, and anti-cancer effects [7,8].

Flax sprouts have garnered significant attention among researchers. Previous scientific research has shown that germination could be an efficient method for accumulating phytochemical profiles in flax. Additionally, ultrasonic pretreatment could affect the content of metabolite levels (including lignan, tocopherol, tocotrienol, and carotenoid) in flax sprouts, offering potential applications for the cultivation of high-value flax [9]. Though the effect of germination and abiotic stress on the bio-compound accumulation during flaxseed development is well studied, there is limited information on the evaluation of the nutritional quality of flaxseed germplasm, especially concerning micronutrients such as polyphenols and vitamins [10,11].

It is imperative to comprehend this information, as well as the nutrient composition of flaxseeds, which could contribute to the high-value utilization of flaxseeds. This study was carried out to evaluate the variations in characteristics, lignan content, and vitamins levels among 40 different varieties of flaxseeds while exploring the internal relationship between varieties with nutrient compositions.

## 2. Materials and Methods

### 2.1. A Collection of 40 Varieties of Flaxseed

Forty flaxseed (*Linum usitatissimum* L.) cultivars were used in this study: Argentin 2195, F2004035, BETHUE, F20070121, YJ005721, YJ005721, F2010083, F2004024, F20070004, F2004012, F2004013, F2004035, F2004037, F2004045, F2004047, F2004052, USPEA, r8607-4-1-3, 89156-12-10-14, baxuan3, dingya22, tongya10, shuangya12, 0407-8-14, siziwangqi, lunxuan3, 2012GYSTS, 2012GYFX-16, 0325-2-2-8, Y4S164-2-25-16, 0419-2-4, 0419-2-8, 0420-2-9, 0325-30-22-7, 0325-3-9-5, 0325-2-26-5, 0325-3-32-9, Y5S038-10-4, Y5S038-10-2, Y4S185-7-9, and Y5S038-10-4. The cultivars were obtained from the Institute of Bast Fiber Crops and Center of Southern Economic Crops, Chinese Academy of Agricultural Sciences (Changsha, China). Prior to the study, over 600 varieties were planted in Yunnan Province, and 40 flaxseed cultivars were selected because of their market potential. In addition, these cultivars were assigned numerical names for ease of reference. Table 1 provides a comprehensive description of each cultivar’s agronomic characteristics including accession name, petal color, type, fiber content in straw, seed yield, vegetation period, and plant natural height. 

### 2.2. Extraction and Analysis of Lignan

The extraction and analysis of lignan in flaxseeds was conducted based on a previously reported method, with some modifications [12,13]. Briefly, the seed samples were ground at −80 °C in liquid nitrogen using a basic mill. Subsequently, 20 mM NaOH dissolved in 50% methanol was used for sample integration. After shaking on a digital rotator for over 12 h, the mixture was neutralized to pH 7.0 via acetic acid and mixed with hexane in order to remove the lipid. Following the evaporation of methanol from the mixture, the extracts was reconstituted in 2 mL purified water, which was stored at −40 °C for further analysis.

Lignan analysis was conducted using a water series high-performance liquid chromatography (HPLC) system (Waters 2695, Waters Corporation, Milford, MA, USA) equipped with an injector (model 2707), a detector (Waters 2998 PDA), and a RP18 column. Lignan was quantified as SDG and SECO (secoisolarisiresinol diglucoside and secoisolarisiresinol, respectively, Santa Ana, CA, USA) [12]. 

### 2.3. Extraction and Analysis of Tocopherol, Tocotrienols and Carotenoids

Extraction and quantification of tocopherol, tocotrienols, and carotenoids were performed following a previously reported method [9]. In brief, flaxseeds were powdered and mixed with ethanol, sodium chloride, and pyrogallol in ethanol and ascorbic acid. The organic layer of the samples was obtained for evaporation using nitrogen gas. Residues were separated and dissolved in hexane solution with isopropyl alcohol (1%) for tocopherol and tocotrienol detection or in ethyl acetate for carotenoid detection, respectively. Sample were stored at −40 °C for prior HPLC analysis.

Tocopherol and tocotrienol analysis was performed via a Waters series HPLC system equipped with a 2475 multi-fluorescence detector and a 515 pump. Chromatographic analyses were carried out with a Zorbas RX-SIL column (4.6 × 250 mm, 5 μm, Agilent Technologies, Santa Clara, CA, USA). Tocopherol and tocotrienol were detected at the excitation wavelength of 290 nm and the emission wavelength of 330 nm, respectively, which were identified and quantified using standard tocopherol (Wako Pure Chemical Industries, Tokyo, Japan) and tocotrienol (ChromaDex, Ltd., Irvine, CA, USA) isomers. In our study, carotenoid analysis was carried out via HPLC-UV employing an Agilent 1260 Infinity II LC system. A carotenoid 30 column (4.6 × 250 mm, 5 μm, YMC Co. Ltd., Komatsu, Japan) was utilized for chromatographic analyses. Mobile phases consisted of 0.1% (*w*/*v*) 2,6-Di-tert-butyl-4-methylphenol (BHT) and 0.05 M ammonium acetate in 97% (*w*/*v*) methanol–water (phase A) and 0.1% (*w*/*v*) BHT in methyl tert-butyl ether (phase B). The flow rate was set as 1 mL/min, and the UV absorbance was 450 nm. Carotenoids were quantified using standard carotenoid (CaroteNature, Erlenauweg, Switzerland) [7,9]. 

### 2.4. Statistical Analysis

In this study, IBM SPSS 25.0 (SPSS Inc., Chicago, IL, USA) was used to analyze the variance among different flaxseeds via a one-way analysis of variance (ANOVA). Statistical significance was determined at *p* < 0.05. The frequency, principal component analysis, and Pearson’s correlation analysis were carried out with Origin 2021 [14].

## 3. Results and Discussion

### 3.1. Lignans in Flaxseed of 40 Selected Varieties

In this study, lignans were identified and quantified in the 40 selected varieties of flaxseeds, with the detailed data in Supplemental Table S1. The results indicated the absence of SECO in numerous flaxseed varieties, while varying levels of SDG were present in all varieties [15]. The final step in SDG biosynthesis within flaxseed involves SECO glycosylation. As the test sample consists of dry seeds, the reduced water content may facilitate SECO glycosylation, leading to the conversion of SECO into SDG and potentially other forms of lignans that cannot be detected, resulting in a decrease in detectable SECO content [16]. Furthermore, as a stable and predominant form of lignan, SDG is widely regarded as equivalent with lignan [17]. The highest contents of SDG were observed in No. 225 (2505.76 ± 14.75 mg/100 g DW) and No. 221 (2318.99 ± 165.67 mg/100 g DW), while the lowest were found in No. 299 (218.54 ± 1.71 mg/100 g DW). The SDG content of the first two varieties of flaxseeds is nearly 11 times that of variety 299. However, the range of content variation in variety 225 is smaller, indicating that this variety has stronger stability. The average of SDG content was 710.31 mg/100 g DW. There is a huge difference in the content of SDG among different varieties, indicating that there is a correlation between its content and variety. Furthermore, the considerate variation in SDG content observed among different flaxseeds suggests that agronomic characteristics play a significant role in determining lignan levels [17].

As shown in Table 2, the average values of SDG in three types of flaxseeds exhibited significant differences. The SDG concentration in flaxseed of fiber flax was higher than in other types [18], indicating that the flaxseed of fiber flax might have higher lignan levels in contrast to other types of flaxseeds. This may be due to the enhanced expression of SDG biosynthetic genes in fiber flax, resulting in higher levels of SDG content compared to oil flax or intermediate flax. Further analysis and discussion are needed to confirm this through gene expression. However, since different types of flaxseeds were obviously different in SDG content (CV = 81.50%), it was implied that even within the same type, SDG content also have significant differences. Therefore, flax type might not a key factor for lignan accumulation in flaxseeds. The present result was similar to that of a previous study, which showed the SDG content of oil and fiber flax was 25.81 and 5.09 mg/g DW, respectively, also showing that flax type was not a crucial factor for the lignan compounds in germinated flax. In a word, it is suggested that No. 217, No. 58, No. 221, and No. 225 could be applied as attractive sources of lignan for further utilization.

### 3.2. Tocopherols and Tocotrienols in 40 Selected Varieties of Flaxseed

The comprehensive characterization of tocopherols and tocotrienols in 40 varieties of flaxseeds are presented in Supplemental Table S2. The main components of vitamin E are tocopherol and tocotrienol. Six isomers were detected in these samples, including α-tocopherol (α-T), α-tocotrienol (α-T3), β-tocopherol (β-T), γ-tocopherol (γ-T), γ-tocotrienol (γ-T3), and δ-tocopherol (δ-T). The total vitamin E content was calculated as the sum of the contents of six isomers. Table 3 shows that the content of γ-T is significantly higher than that of γ-T3, and γ-T was the highest among tocopherols and tocotrienols, which took up 53.25%. It is stated that the vitamin E in dry flaxseeds is mainly composed of tocopherols, with γ-T being the main component, which is consistent with previous research findings. However, their specific content is influenced by both variety and environment [19]. The average γ-T content of 40 varieties of flaxseed was 410.18 μg/g DW, and the CV was 93.17%, which suggested that these flaxseeds had a wide range of γ-T content [9]. Tocopherol cyclase and γ-tocopherol methyl transfer enzyme are important enzymes in the biosynthetic pathway of vitamin E, determining the accumulation of tocopherol types in plants. Tocopherol cyclase is involved in the formation of γ- and δ-Ts [20]. The high content of γ-T may be due to higher activity of tocopherol cyclase, resulting in the greatest accumulation during γ-T biosynthesis. However, enzyme activity may vary within different varieties, leading to a wider range of variations. The highest content was found in No. 549, while the lowest was observed in No. 627. Notably, β-T also had a high CV (91.80%), which implied that these flaxseed samples had significant difference in β-T, which had the highest observed values in No. 167. In addition, No. 2, 611, 315, 603, 299, 148, 167, 16, and 549 had high contents of β-T, which was about 2.50-fold higher than the lowest level. The total VE content, which was calculated as the sum of all tocopherols and tocotrienols, was analyzed in our study. The average total VE content among 40 flaxseeds was 1025.18 μg/g DW, which was similar with the results of a previous report [7,8]. However, in comparison to the flax oil used in the other study, our seeds exhibited a relatively lower total VE content [5,21,22], suggesting that different flaxseeds might have differences in VE because of their origin, as well as the type of flax [23,24].

### 3.3. Carotenoids in Selected 40 Varieties of Flaxseeds

The nutritional value as well as the metabolite levels of these 40 varieties of flaxseeds were investigated in this study to elucidate the presence of carotenoids, including lutein (Lut.), β-carotene (β-Car.), and zeaxanthin (Zea.). The total carotenoid (Total Car.) content was determined by summing the contents of these three isomers [25]. The detailed description of carotenoids in 40 varieties of flaxseeds is exhibited in Supplemental Table S3. Among all the varieties, variety No. 16 exhibited the lowest Lut., Zea., β-Car., and total Car. and the largest content for carotenoid compositions that were not easy to observe directly. For Lut., the content ranged from 36.29 to 152.36 μg/100 g DW, with the highest content found in No. 168. The content for Zea. ranged from 2.99 to 21.96 μg/100 g DW, and the highest content was found in No. 217. For β-Car., values ranged from 5.92 to 52.03 μg/100 g DW, and the highest content was found in variety No. 167. In previous studies on quantitative analysis of carotenoids in flaxseeds, no β-carotene was found, which may be related to the selection of flaxseed varieties. The total Car. ranged from 45.2 to 310.84 μg/100 g DW, and the highest content was found in No. 164. Then, this study divided flax types into fiber flax, oil flax, and intermediate flax for further analysis. Table 4 shows that Lut. had the highest content among the carotenoids, which took up 70.08, 71.17, and 80.28%, respectively. Flaxseeds contain abundant lutein, which means they are more resistant to storage compared to fruits and can be an excellent source of carotenoids. The average Lut. content of fiber flax was 86.03, and the CV was 36.80%, which suggests that these flaxseeds had a wide range of Lut. content [10]. The average Lut. content of oil flax was 96.34, with a CV of 43.99%. The average Lut. content of intermediate flax was 36.29, and the CV was 7.39%, indicating a narrow range in Lut. content for these flaxseeds. The content of Zea. was the lowest among the carotenoids, making up 9.95, 12.56, and 2.99%, respectively. The average Zea. content of fiber flax was 9.95, and the CV was 44.39%. The average Zea. content of oil flax was 12.56, and the CV was 37.25%, which suggests that these flaxseeds had a wide range of Zea. content. The average Zea. content of intermediate flax was 2.99, and the CV was 3.09%, which suggests that these flaxseeds had a narrow range of Zea. content. For all varieties of flax, the average content of Lut. was 89.42, and the CV was 42.16%. The average content of Zea. was 10.95, and the CV was 43.99%. The average content of β-Car. was 26.14, and the CV was 57.01%. These values suggest that these flaxseeds had a wide range of carotenoid contents [26]. The color of food is a very important factor in determining its acceptability [27]. The study of Cui et al. indicated that the average dynamic viscosity of gums, Foster, and Omega from selected yellow-seeded cultivars was higher than that of gums extracted from brown seed [28]. However, there was no apparent correlation been found between carotenoids and the color of flaxseeds, which contradicts previous research [5,26]. This discrepancy may be attributed to differences in crop varieties.

### 3.4. Frequency Distribution Analysis of Phytochemicals in 40 Varieties of Flaxseeds

**Figure 1:**
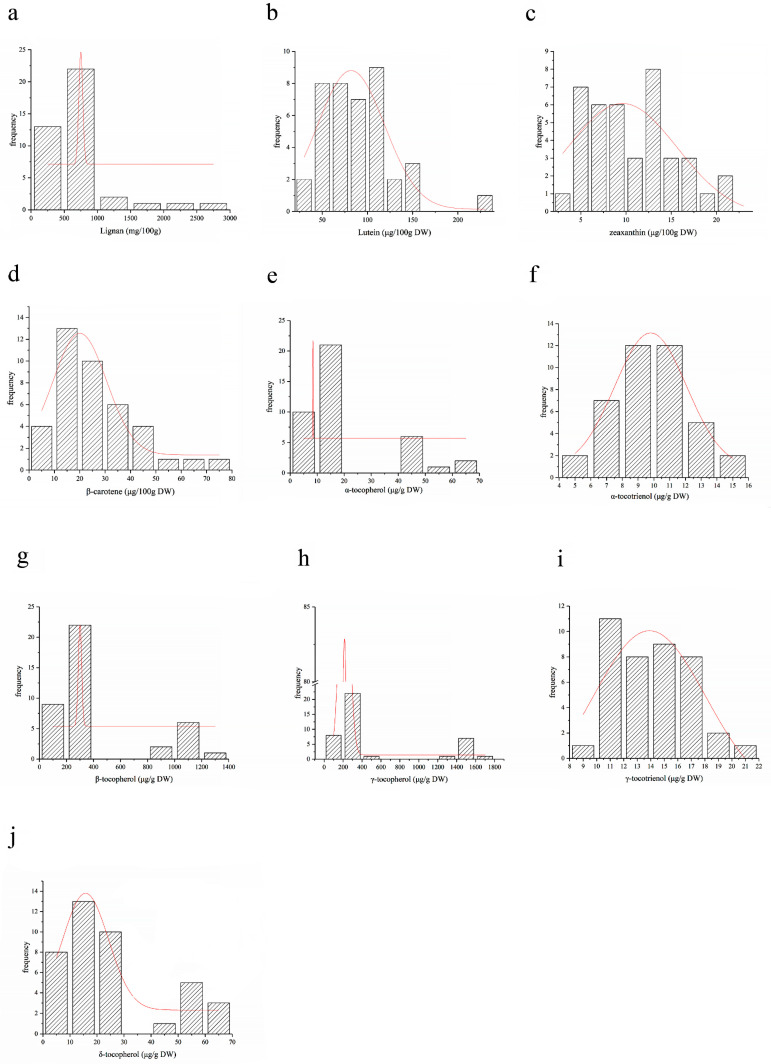
(**a**–**j**) Frequency distribution analysis of phytochemicals in 40 varieties of flaxseeds.

The frequency distribution analysis of the phytochemicals in the 40 varieties is presented in Figure 1. In terms of lignan, the majority of concentrations ranged between 500–1000 mg/100 g DW, with more than 20 varieties falling within this interval (Figure 1a). Lutein (Lut.) was mainly concentrated between 50–150 mg/100 g DW; more than 32 varieties were located in this range (Figure 1b). Zeaxanthin (Zea.) was mainly concentrated between 5–20 mg/100 g DW; more than 37 varieties were located in this range (Figure 1c). β-carotene (β-Car.) was predominantly concentrated between 10–50 mg/100 g DW; more than 34 varieties were located in this range (Figure 1d). α-tocopherol (α-T) was mainly concentrated between 10–20 μg/g; more than 22 varieties were located in this range (Figure 1e). α-tocotrienol (α-T3) was mainly concentrated between 7–13 μg/g; more than 36 varieties were located in this range (Figure 1f). β-tocopherol (β-T) was primarily concentrated between 200–400 μg/g; more than 22 varieties were fell into this category (Figure 1g). γ-tocopherol (γ-T) was mainly concentrated between 0–400 μg/g; more than 29 varieties were located in this range (Figure 1h). For γ-tocotrienol (γ-T3), more than 36 varieties were between 10–18 μg/g (Figure 1i). δ-tocopherol (δ-T3) was mainly concentrated between 0–30 μg/g; more than 31 varieties were located in this range (Figure 1j). Overall, the differences in nutrient composition among the cultivars were enormous.

### 3.5. Principle Components Analysis (PCA)

**Figure 2:**
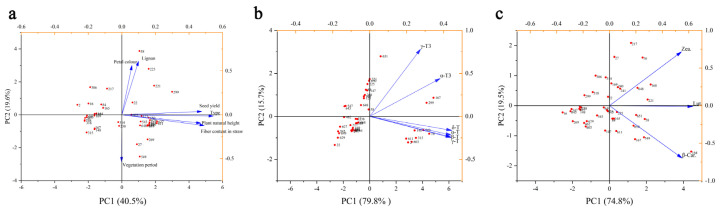
PCA analysis in varieties. (**A**) PCA analysis between agronomic traits and varieties. (**B**) PCA analysis between vitamins and varieties. (**C**) PCA analysis between carotenoids and varieties.

The PCA analysis of agronomic traits and varieties is shown in Figure 2. Principal component analysis of the agronomic traits indicates that the variance contribution rates of PC1 and PC2 are 40.5 and 19.6%, respectively, with a cumulative variance contribution rate reaching 60.1%. The absolute values of the characteristic vector values for grain yield, variety, plant height, and straw fiber content are large, indicating that PC1 is determined by grain yield, variety, plant height, and straw fiber content. Similarly, the absolute values of the characteristic vector values for lignin and petal color are large, indicating that PC2 is determined by lignin and petal color. This suggests that agronomic traits can be comprehensively evaluated based on grain yield, variety, plant height, straw fiber content, lignin, and petal color. From the graph, it is evident that there exists a significant positive correlation between grain yield, variety, plant height, and straw fiber content. Moreover, lignin exhibits a highly positive correlation with petal color. The duration of vegetation demonstrates a noteworthy negative correlation with both petal color and lignin. Although it displays a negative correlation with seed yield, the association is not statistically significant (Figure 2a). The PCA of vitamins showed that the variance contribution rates of PC1 and PC2 are 79.8% and 15.7%, respectively, with a cumulative variance contribution rate reaching 95.5%. This indicates that these two principal components can be used for comprehensive evaluation of vitamins. The absolute values of the characteristic vector values for four types of tocopherols are large, indicating that PC1 is determined by these four types of tocopherols, while PC2 is determined by two types of tocotrienols. This suggests that vitamin E can be comprehensively evaluated based on the combination of these four types of tocopherols and two types of tocotrienols (Figure 2b). There is a highly significant correlation among four types of tocopherols and a significant correlation between two types of tocotrienols. There is also some correlation between tocopherols and tocotrienols. The principal component analysis of carotenoids showed that the contribution rates of PC1 and PC2 were 74.8% and 19.5%, respectively, with a cumulative variance contribution rate reaching 94.3%. PC1 was determined by three substances collectively, while PC2 was determined by zeaxanthin (Zea.) and β-carotene (β-Car.), indicating that carotenoids can be comprehensively evaluated with these substances. Lutein (Lut.) revealed a strong correlation with zeaxanthin (Zea.) and β-carotene (β-Car.). Zeaxanthin (Zea.) showed a non-significant correlation with β-carotene (β-Car.) (Figure 2c).

## 4. Conclusions

In this study, the levels of lignan, vitamins, and carotenoids were determined in 40 selected varieties. Among the different flax types, the seeds of intermediate flax exhibited the lowest content and the seeds of fiber flax had the highest content. For tocopherol and tocotrienol, the seeds of intermediate flax showed the highest content. The seeds of fiber flax had the lowest content of α-T, β-T, and γ-T. The seeds of oil flax had the lowest content of γ-T3, while no significant difference was observed in α-T3 levels. For carotenoids, the seeds of intermediate flax had the lowest content in all species. The seeds of oil flax had the highest content in Zea. and total Car. The seeds of fiber flax had the highest content of Lut. There appears to be a certain relationship between the type and nutrients composition. However, further exploration is required to fully understand the internal relationship. There exists a significant positive correlation among seed yield, variety, plant natural height, and fiber content in straw, which can be utilized for the preliminary selection of high-yielding and high-quality plants. The relationship between lignan and petal color also demonstrates a significant positive correlation, enabling the determination of lignan levels through petal coloration to produce valuable products. Both four types of tocopherols content and two types of tocotrienols exhibit significantly positive correlations, providing a comprehensive evaluation approach for vitamin E. Flaxseeds present higher resistance to carotenoids compared to fruits and vegetables, thus serving as an excellent alternative source. The research results show that there are varying levels of lignans and trace nutrients in 40 varieties of flaxseeds, which means that flaxseeds may be developed as a resource for health products in the future or applied in the functional food industry. These findings will lay the foundation for studying the nutritional components of flaxseed grains and provide a basis for developing and utilizing high-value products from flax.

## Figures and Tables

**Table 1:** Description of agronomic characteristics, including accession name of number, petal color, type, fiber content in straw, seed yield, vegetation period, and plant natural height, of 40 varieties of flaxseed (*Linum usitatissimum* L.).

Varieties	Accession Name or Number	Petal Color	Type	Fiber Content in Straw (%)	Seed Yield (kg/ha)	Vegetation Period (d)	Plant Natural Height (cm)
2	Argentin 2195	light blue	linseed	9.17%	2119.50	159	63.92
16	F2004035	white	intermediate	11.28%	2073.50	126	60.35
20	BETHUE	light blue	linseed	6.25%	2360.00	128	64.32
22	F20070121	light blue	flax	14.55%	1443.00	152	91.95
27	YJ005721	light blue	flax	22.17%	2067.50	145	79.15
58	F2010083	blue	flax	13.84%	1281.50	152	90.03
84	F2004024	light blue	linseed	12.10%	1994.50	152	74.18
121	F20070004	light blue	flax	20.24%	1529.25	149	90.08
147	F2004012	light blue	linseed	12.87%	2385.25	151	70.05
148	F2004013	light blue	linseed	12.39%	2296.75	142	59.42
164	F2004035	white	linseed	12.10%	2343.50	141	70.12
165	F2004037	light blue	linseed	13.45%	2194.75	144	72.23
167	F2004045	light blue	linseed	12.11%	2369.00	141	68.79
168	F2004047	light blue	linseed	9.93%	2365.00	145	70.16
169	F2004052	light blue	linseed	12.57%	2518.00	150	68.32
217	USPEA	white	linseed	16.96%	2500.00	148	68.58
221	r8607-4-1-3	light blue	flax	15.05%	978.25	148	93.85
225	89156-12-10-14	white	flax	16.54%	1653.75	145	106.81
238	baxuan3	light blue	linseed	15.94%	2066.50	149	77.43
240	dingya22	light blue	linseed	13.11%	2201.75	151	70.15
241	tongya10	light blue	linseed	12.63%	1995.50	151	70.76
269	shuangya12	light blue	flax	19.08%	1007.00	140	91.68
299	0407-8-14	violet	flax	22.00%	1193.00	151	83.76
306	siziwangqi	blue	linseed	10.22%	2246.25	150	54.26
314	lunxuan3	light blue	linseed	13.62%	2400.00	151	76.63
315	2012GYSTS	light blue	linseed	14.80%	2024.75	142	55.26
318	2012GYFX-16	light blue	linseed	6.17%	2600.00	147	62.74
462	0325-2-2-8	light blue	flax	22.21%	1612.00	147	90.27
543	Y4S164-2-25-16	light blue	flax	19.91%	1623.50	148	90.46
547	0419-2-4	light blue	flax	20.43%	1466.00	149	101.19
549	0419-2-8	light blue	flax	18.92%	1452.50	159	92.16
603	0420-2-9	light blue	flax	8.78%	961.25	149	98.86
611	0325-30-22-7	light blue	flax	20.71%	1102.75	149	100.72
627	0325-3-9-5	light blue	flax	25.91%	1305.50	148	93.28
629	0325-2-26-5	light blue	flax	22.98%	1229.75	150	95.82
643	0325-3-32-9	light blue	flax	21.75%	1053.75	148	89.65
648	Y5S038-10-4	light blue	flax	18.30%	1121.00	149	101.27
649	Y5S038-10-2	light blue	flax	15.06%	993.00	150	95.64
650	Y4S185-7-9	light blue	flax	15.84%	956.00	148	99.97
651	Y5S038-10-4	light blue	flax	16.83%	1041.25	150	93.79

**Table 2:** Statistic data of lignan (mg/100 g DW) in 40 varieties of flaxseeds.

Flax Type	Component	Average	Stdev	CV/%	Percent of Total Phytochemical Content/%
Flax	Secoisolarisiresinol diglucoside	774.48	631.19	81.50	/
Linseed		646.38	265.91	41.14	/
Intermediate		513.58	19.67	3.83	/
Total		710.31	494.04	69.55	/

**Table 3:** Statistical data of tocopherol and tocotrienol (μg/g DW) in 40 varieties of flaxseeds. The ‘T’ in ‘α-T’, ‘β-T’, ‘γ-T’, and ‘δ-T’ stands for ‘tocopherol’, while ‘T3’ stands for ‘tocotrienol’.

Flax Type	Component	Average	Stdev	CV/%	Percent of Total Phytochemical Content/%
Flax	α-T	17.03	15.33	90.02	1.87
	α-T3	9.65	3.26	33.81	1.06
	β-T	356.37	338.29	94.93	39.11
	γ-T	491.73	519.48	105.64	53.97
	γ-T3	14.74	4.39	29.76	1.62
	δ-T	21.62	18.46	85.35	2.37
Linseed	α-T	20.14	16.39	81.37	1.90
	α-T3	9.97	2.12	21.28	0.94
	β-T	429.98	377.91	87.89	40.65
	γ-T	556.51	452.77	81.36	52.61
	γ-T3	13.60	3.42	25.13	1.29
	δ-T	27.57	15.98	57.97	2.61
Intermediate	α-T	64.16	3.17	4.94	2.26
	α-T3	13.70	1.18	8.60	0.48
	β-T	1184.09	53.54	4.52	41.79
	γ-T	1491.67	56.38	3.78	52.65
	γ-T3	15.53	1.07	6.87	0.55
	δ-T	64.02	1.45	2.27	2.26
Total	α-T	19.61	17.21	87.73	1.91
	α-T3	9.90	2.82	28.54	0.97
	β-T	410.18	374.29	91.25	40.01
	γ-T	545.88	506.08	92.71	53.25
	γ-T3	14.25	3.95	27.73	1.39
	δ-T	25.36	18.40	72.56	2.47

**Table 4:** Statistical data of carotenoids in 40 varieties of flaxseeds. The ‘Lut.’ stands for ‘Lutein’, ‘β-Car.’ stands for ‘Carotene, ‘Zea.’ stands for ‘zeaxanthin’, and ‘Total Car.’ stands for ‘Total carotenoids’.

Flax Type	Component	Average	Stdev	CV/%	Percent of Total Phytochemical Content/%
Flax	Lut.	86.03	31.66	36.80	70.08
	Zea.	9.95	4.42	44.39	8.11
	β-Car.	26.78	14.04	52.43	21.81
	Total Car.	122.75	46.67	38.02	/
Linseed	Lut.	96.34	42.38	43.99	71.14
	Zea.	12.56	4.68	37.25	9.27
	β-Car.	26.52	15.63	58.93	19.58
	Total Car.	135.42	56.94	42.05	/
Intermediate	Lut.	36.29	2.68	7.39	80.28
	Zea.	2.99	0.09	3.09	6.62
	β-Car.	5.92	0.30	5.01	13.10
	Total Car.	45.20	3.03	6.70	/
Total	Lut.	89.42	37.70	42.16	70.68
	Zea.	10.95	4.82	43.99	8.65
	β-Car.	26.14	14.90	57.01	20.66
	Total Car.	126.52	52.81	41.74	/

## Data Availability

Data is unavailable due to privacy or ethical restrictions.

## Supplementary Materials

The following supporting information can be downloaded at: https://www.mdpi.com/article/10.3390/foods12234250/s1, Table S1: The contents of lignan in 40 varieties of flaxseeds (mg/100 g DW); Table S2: The contents of vitamin E isomers in 40 varieties of flaxseeds (μg/g DW). The ‘T’ in ‘α-T’, ‘β-T’, ‘γ-T’ and ‘δ-T’ stands for ‘tocopherol’ while ‘T3’ stands for ‘tocotrienol’; Table S3: The contents of carotenoid compositions in 40 varieties of flaxseeds (μg/100 g DW). ‘Lut.’ stands for ‘Lutein’, ‘Zea’ stands for ‘Zeaxanthin’, the ‘Car.’ in ‘β-Car.’ stands for ‘carotene’; Figure S1: Typical structures of representative metabolites. (a) lignans. (b) tocopherol. (c) tocotrienols. (d) carotenoids.
