# Supplementary material for: Comparative Assessment of Lignan, Tocopherol, Tocotrienol and Carotenoids in 40 Selected Varieties of Flaxseed (Linum usitatissimum L.)
Source: Foods. 2023 Nov 24;12(23):4250. doi: 10.3390/foods12234250 (PMC10706792; doi:10.3390/foods12234250)
Supplement: Supplementary file 1 [file foods-12-04250-s001.zip › foods-2660832-supplementary.pdf]

Supplemental Table S1. The contents of lignan in 40 varieties of flaxseeds (mg/100g DW).

Varieties	SDG
2	373.36±9.36bcd
16	513.58±19.67fgh
20	430.97±9.02def
22	674.84±23.03jklmn
27	362.06±15.36bcd
58	1869.36±135.89s
84	434.86±17.07def
121	849.77±16.6p
147	737.42±39.98mno
148	708.82±42.57lmn
164	820.54±77.83op
165	1003±100.14q
167	755.3±65.04nop
168	326.71±26.94bcd
169	648.95±9.49ijklmn
217	1415.52±138.6r
221	2318.99±165.67t
225	2505.76±14.75u
238	514.33±32.41fgh
240	605.08±57.9hijkl
241	688.6±27.19klmn
269	401.63±9.42cde
299	218.54±1.71a
306	737.11±63.47mno

314	565.84±56.28ghi
315	582.84±0.05ghijk
318	285.51±27.76ab
462	575.2±54.15ghij
543	572.07±38.78ghij
547	495.23±44.82efg
549	319.91±20.31bc
603	678.05±51.63jklmn
611	564.51±34.29ghi
627	656.4±48.29ijklmn
629	748.37±45.53mno
643	639.45±62.97ijklm
648	382.69±36.39bcd
649	363.73±7.24bcd
650	733.51±72.37mno
651	334.04±13.53bcd
Average/ (mg/g DW)	710.31
Stdev	494.04
CV%	69.55

The difference of letter indicates a significant difference between values ($p < 0.05$).

Supplemental Table S2. The contents of vitamin E isomers in 40 varieties of flaxseeds (μg/g DW). The 'T' in 'α-T', 'β-T', 'γ-T' and 'δ-T' stands for 'tocopherol' while 'T3' stands for 'tocotrienol'.

Varieties	α-T	α-T3	β-T	γ-T	γ-T3	δ-T	total VE
2	47.23±2.21m	11.6±0.98hijkl	1036.78±33.96jk	1288.68±55.31f	13.62±1.1abcd	51.58±2.41k	2449.48±91.64f
16	64.16±3.17p	13.7±1.18lm	1184.09±53.54l	1491.67±56.38gh	15.53±1.07abcde	64.02±1.45m	2833.18±110.56ij
20	15.72±2.01jk	8.63±1.64abcdefgh	223.09±39.88def	332.66±21.07d	12.85±1.93abcde	20.45±0.96cdefgh	613.39±62.26bcd
22	6.65±0.32abc	5.3±0.48a	136.73±12.94a	128.53±11.61a	9.95±2.42a	5±0.45a	292.15±27.28a
27	10.17±0.07def	9.47±1.2cdefghi	226.26±3.61def	294.5±8.99cd	12.65±2.4abc	18.97±1.19bcdefg	572.03±11.67bc
58	18.09±0.82k	10.75±0.78efghijl	332.09±29.69i	436.28±33.46e	14.4±3.11abcde	25.2±0.29i	836.83±66.27e
84	12.43±0.72fgh	11±0.91ghijkl	268.57±26.55efgh	337±31.83d	16.7±1.26abcde	17.91±1.94bcd	663.61±59.71bcd
121	11.19±1.31defgh	10.98±2.15fghijl	221.9±34.74def	347.26±45.91de	15.26±3.24abcde	21.19±0.27fgh	627.78±83.65bcd
147	13.49±0.86ghij	11.33±5.3ghijkl	274.98±10.4fgh	366.78±3.19de	16.5±8.76abcde	18.52±0.8bcdef	701.59±24.38cd
148	46±3.5m	11.25±1.14ghijkl	1170.01±77.09l	1432.8±91.37g	16.34±1.19abcde	52.77±4.11k	2729.16±176.16hi
164	13±1.33fghij	8.67±1.65abcdefgh	243.52±15.1defgh	332.71±29.85d	11.55±3.28abc	21.87±1.86gh	631.32±50.34bcd
165	12.67±0.46fghi	10.02±1.61defghij	247.99±7.32defgh	328.59±3.94cd	16.25±1.19abcde	21.08±1.79efgh	636.61±11.58bcd

167	60.33±4.35o	14.17±2.5lm	1239.55±16.13m	1408.05±28.63g	20.73±4.32e	57.25±1.04l	2800.08±43.84ij
168	11.24±0.32defgh	9.59±1.13cdefghi	295.58±21.16hi	320.34±16.61cd	12.33±0.74abc	22.24±0.3h	671.32±36.17bcd
169	10.93±0.32defg	8.56±0.48abcdefgh	239.13±6.04defgh	308.27±7.94cd	11.79±1.01abc	17.47±0.59bc	596.14±13.32bcd
217	10.59±0.15defg	8.76±0.35bcdefgh	135.68±6.32a	228.31±5.05bc	11.7±0.64abc	17.09±1.17b	412.14±8.9a
221	15.49±0.03ijk	11.99±0.31ijklm	201.67±20.22bcd	312.75±23.64cd	17.8±0.75cde	19.92±1.36bcdefgh	579.61±42.5bcd
225	12.05±0.75efgh	11.24±2.2ghijkl	238.72±15.05defgh	342.19±14.22de	17.46±4.4bcde	19.99±0.97bcdefgh	641.66±9.37bcd
238	12.5±0.62fgh	9.44±0.83cdefghi	230.74±16.12defg	328.67±15.89cd	12.84±1.46abcd	19.66±0.19bcdefgh	613.84±30.79bcd
240	9.32±4.87cde	8.52±1.23abcdefgh	208.08±16.3bcde	292.95±2.18cd	11.27±0.65abc	18.54±1.15bcdef	548.69±22.99b
241	10.94±0.9defg	8.44±0.59abcdefgh	206.51±16.09bcde	316.77±24.48cd	11.27±0.94abc	18.08±0.65bcde	572.02±39.79bc
269	10.37±0.37def	11±3.09ghijkl	250.15±6.61defgh	357.27±12.76de	15.82±5.07abcde	20.83±0.91defgh	665.45±20.45bcd
299	47.26±0.38m	13.32±1.42klm	1076.06±84.67k	1483.96±61.07gh	19.83±8.02de	63.79±1.52m	2704.21±117.17hi
306	8.99±0.42cd	8.97±0.92bcdefghi	256.5±1.25defgh	296.41±6.12cd	11.23±0.53abc	17.67±0.26bc	599.76±7.12bcd
314	11.33±0.91defgh	9.06±0.22bcdefghi	216.24±6.92def	305.49±8.77cd	11.1±0.46abc	17.99±0.42bcde	571.2±16.42bc
315	45.41±2.44m	12.33±1.35jklm	1039.65±96.22jk	1431.76±99.09g	14.48±1.81abcde	64.67±4.4m	2608.3±200.85gh
318	10.49±0.48defg	9.2±0.1bcdefghi	207±9.49bcde	361.02±20.92de	12.25±1.04abc	21.38±1.13fgh	621.34±22.45bcd

462	5.53±0.01a	7.5±3.07abcdef	152.79±13.25abc	154.03±11.94ab	13.17±4.95abcd	6.19±0.21a	339.22±18.97a
543	5.65±1.25a	6.24±1.18abc	121.83±9.45a	133.36±7.07ab	11.49±3.25abc	4.74±0.06a	283.32±21.15a
547	5.87±0.27ab	7.28±0.15abcde	143.13±6.78a	139.17±12.8ab	15.08±0.87abcde	5.12±0.05a	315.66±16.77a
549	52.64±0.62n	13.44±0.94klm	1092.62±34.39k	1667.8±232.18i	15.66±1.47abcde	56.74±2.91l	2898.9±214.25j
603	41.61±0.63l	11.85±1.5hijklm	984.55±43.27j	1545.06±72.91h	13.87±1.57abcde	59.46±2.81l	2656.4±122.45h
611	47.9±1.25m	11.42±1.01hijkl	988.37±47.77j	1403.75±63.69g	14.17±1.45abcde	41.83±3.06j	2507.44±116.68fg
627	5.87±0.43ab	6.84±0.89abcd	138.52±10.82a	116.07±10.36a	11.9±2.58abc	4.89±0.43a	284.08±19.8a
629	6.83±0.24abc	6.51±0.55	130.41±12.68a	123.29±19.24a	10.37±0.29ab	5.49±0.25a	282.91±32.71a
643	5.83±0.48ab	6.31±0.84abc	149.24±9.48ab	138.43±18.9ab	15.63±4.42abcde	5.31±0.42a	320.75±28.33a
648	14.01±1.06hij	7.85±0.65abcdefg	262.8±14.41defgh	369.04±38.29de	16.4±5.27abcde	21.75±1.8gh	691.85±31.81bcd
649	8.62±0.31bcd	5.83±0.76ab	136.21±8.03a	142.32±11.63ab	11.64±2.42abc	5.7±0.42a	310.34±15.38a
650	13.01±1.36fghij	12.41±3.57jklm	210.36±32.46cde	325.42±56.49cd	17.17±8.37bcde	19.76±1.19bcdefgh	598.13±101.13bcd
651	13.07±0.14fghij	15.13±3.92m	289.26±14.92ghi	365.83±20.75de	19.79±6.68de	22.21±0.87h	725.29±11.95de

The difference of letter indicates a significant difference between values ($p < 0.05$).

Supplemental Table S3. The contents of carotenoid compositions in 40 varieties of flaxseeds (μg/100g DW). ‘Lut.’ stands for ‘Lutein’, ‘Zea’ stands for ‘Zeaxanthin’, the ‘Car.’ in ‘β-Car.’ stands for ‘carotene’.

Varieties	Lut.	Zea.	β-Car	Total Carotenoid
2	44.53±1.62ab	7.07±0.17fg	14.88±0.18fghi	66.48±1.94bc
16	36.29±2.68a	2.99±0.09a	5.92±0.3a	45.2±3.03a
20	126.91±6.13r	21.53±1.35r	25.3±1.68n	173.73±5.42lmn
22	98.84±2.68lm	10.94±0.38ij	19.76±0.68kl	129.54±3.22i
27	90.25±5.66jk	17.58±0.45pq	12.57±0.51def	120.4±6.13hi
58	143.17±4.69s	13.86±0.57lmn	44.66±3.22r	201.7±7.28o
84	54.8±3.03cd	12.64±0.74kl	40.95±2.01q	108.39±2.31fg
121	95.04±5.92jkl	9.45±0.53h	24.54±2.41mn	129.03±5.04i
147	77.13±6.89hi	7.98±0.59g	34.53±1.26p	119.64±5.34h
148	49.39±2.04bc	6.48±0.31ef	15.09±0.5fghij	70.96±1.71c
164	223.49±1.9u	15.32±1.08o	72.03±0.59v	310.84±0.9p
165	103.23±3.79lmno	9.53±0.47h	30.78±1.7o	143.54±5.68jk
167	110.77±0.16op	11.61±0.5jk	52.03±3.89t	174.41±3.95lmn
168	152.36±4.44t	18.53±0.25q	34.8±2.04p	205.69±6.38o
169	98.14±5.6klm	13.3±0.07lm	17.74±0.21ijk	129.18±5.82i
217	106.09±4.39mno	21.96±1.41r	16.37±1.34hij	144.42±5.31jk
221	143.44±8.93s	16.75±1.32p	39.91±2.57q	200.1±12.36o
225	107.71±4.5no	10.66±1.01hij	29.68±2.12o	148.05±7.34jk
238	65.95±6.4ef	5.74±0.56cde	10.87±0.83bcde	82.57±7.07d
240	107.34±5.6no	13.72±0.89lmn	21.74±0.43lm	142.8±5.13jk
241	116.05±2.29pq	13.49±0.38lmn	21.95±1.99lm	151.49±1.75k
269	39.73±0.7a	4.12±0.34ab	15.77±0.64ghij	59.61±1.63b
299	69.13±7.73fgh	7.7±0.1g	8.51±0.03ab	85.34±7.8de
306	62.38±1.96def	13.04±0.55l	11.61±0.16cde	87.02±2.54de

314	70.03±3.4fgh	14.67±0.81no	17.97±1.18jk	102.67±1.51f
315	94.93±5.54jkl	9.51±0.12h	25.47±0.59n	129.91±5.07i
318	70.64±2.39fgh	9.91±0.58hi	13.3±0.83efg	93.85±1.6e
462	59.99±3.32de	5.13±0.25bcd	20.15±0.74kl	85.28±2.93de
543	74.57±6.62ghi	7.98±0.44g	23.8±0.56mn	106.35±7.07f
547	44.06±2.32ab	4.89±0.18bc	9.54±0.36bc	58.49±2.72b
549	101.76±5.32lmn	14.57±0.32mno	60.14±2.05u	176.46±7.57mn
603	54.48±0.64cd	5.5±0.23cde	23.82±0.82mn	83.81±1.3d
611	88.97±5.92j	9.68±0.15hi	40.24±0.63q	138.89±5.16j
627	51.06±4.09bc	6.25±0.33def	13.65±1.13efgh	70.97±4.9c
629	66.45±3.74efg	5.5±0.48cde	19.99±1.49kl	91.94±5.65de
643	43.02±2.05ab	4.48±0.23bc	9.78±0.18bcd	57.28±2.21b
648	118.8±2.98q	17.47±1.7pq	34.01±0.79p	170.28±3.97lm
649	81.27±6.32i	9.99±0.99hi	24.4±0.96mn	115.66±6.45gh
650	105.23±7.99mno	13.19±0.95l	47.47±3.47s	165.88±10.45l
651	129.55±0.51r	13.28±1.28lm	39.96±3.25q	182.79±3.23n

The difference of letter indicates a significant difference between values ($p < 0.05$).

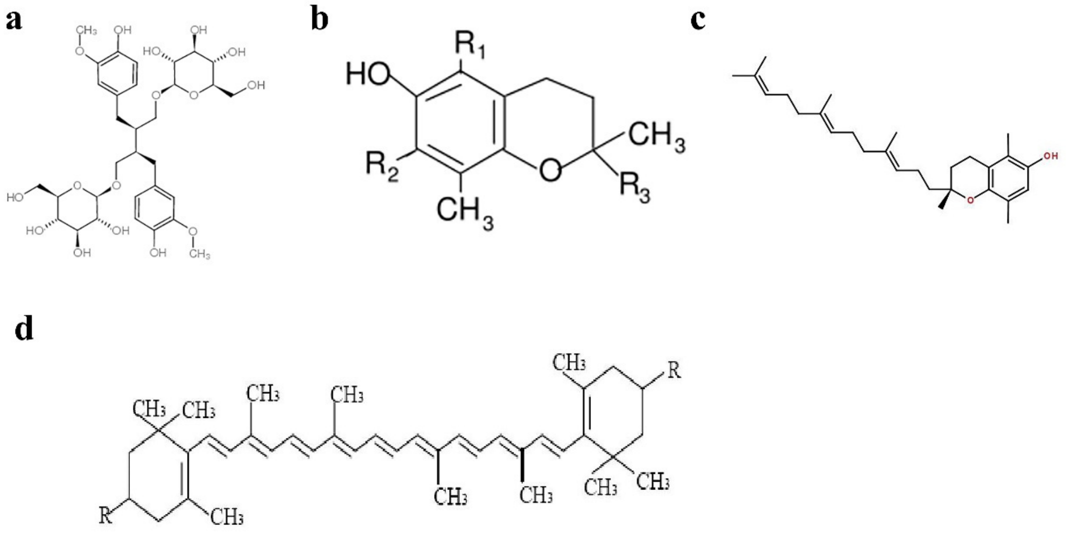

Supplemental Figure S1. Typical structures of representative metabolites. (a) lignans. (b) tocopherol. (c) tocotrienols. (d) carotenoids.
